# Single-cell omics uncovers novel pathological mechanisms and therapeutic targets for congenital heart diseases: insights from integrated intercellular communication analysis

**DOI:** 10.1186/s13287-026-05015-3

**Published:** 2026-04-16

**Authors:** Phuong Thao Nguyen, Makoto Sahara

**Affiliations:** 1 Department of Regenerative Medicine, Hamamatsu University School of Medicine, 1-20-1 Handayama, Chuo-ku, Hamamatsu City, Shizuoka 431-3192 Japan; 2 Department of Surgery, Yale University School of Medicine, 333 Cedar Street, New Haven, CT 06510 USA

**Keywords:** Congenital heart disease, Single-cell RNA sequencing, Therapeutic targets, Intercellular communication, Precision medicine

## Abstract

**Background:**

Congenital heart disease (CHD) affects approximately 1% of live births worldwide and remains the leading cause of infant mortality from congenital anomalies. Despite advances in diagnosis and therapeutics, the molecular mechanisms underlying CHD pathogenesis remain incompletely understood, limiting the development of efficient targeted therapies. Single-cell omics approaches including single-cell RNA sequencing (scRNA-seq) have revolutionized our understanding of cardiac cellular heterogeneity and intercellular signalling. This review synthesizes recent single-cell omics studies in cardiogenesis and CHD, and presents novel insights from an integrated reanalysis to identify potential therapeutic targets.

**Methods:**

We systematically reviewed single-cell omics studies in human cardiogenesis and CHD (2019–2025), then reanalysed the Hill et al. dataset comprising 157,273 nuclei from paediatric patients across five diagnostic categories, including neonatal and infant hypoplastic left heart syndrome (HLHS), tetralogy of Fallot (TOF), dilated cardiomyopathy (DCM) and hypertrophic cardiomyopathy (HCM) with healthy donor controls. We quantified cardiac-specific ligand-receptor interactions to characterize disease-specific intercellular communication networks through pathway enrichment and network topology analysis.

**Results:**

Reanalysis revealed extensive remodelling of cell–cell communication networks across CHD subtypes, each displaying distinct signalling architectures. Neonatal HLHS and TOF demonstrated hyperactivation of metabolic and growth factor pathways with highly centralized endothelial cell (EC) to cardiomyocyte (CM) and cardiac fibroblast (CF) to CM networks. DCM showed selective metabolic enhancement with preserved integration. In contrast, HCM exhibited broad pathway suppression, particularly morphogen signalling, and fragmented connectivity with weakened CF-CM and EC-CM coupling. Infant HLHS represented an intermediate phenotype with suppressed Notch and extracellular matrix signalling.

**Conclusions:**

Single-cell omics studies have revealed cellular heterogeneity and disease-specific mechanisms across CHD subtypes. Our network-based reanalysis demonstrates that CHD involves not only transcriptional defects but also profound disruptions in multicellular communication, with each subtype exhibiting distinct signalling architectures. These findings provide a foundation for precision therapeutic strategies tailored to individual CHD subtypes, with future multimodal approaches accelerating clinical translation.

**Supplementary Information:**

The online version contains supplementary material available at 10.1186/s13287-026-05015-3.

## Introduction

Congenital heart disease (CHD) represents the most prevalent birth defect globally, affecting approximately 8–10 per 1,000 live births and accounting for nearly 30% of all infant deaths due to congenital anomalies [1, 2]. This heterogeneous group of structural cardiac malformations encompasses a spectrum of defects ranging from simple lesions such as small ventricular septal defects (VSDs) to complex malformations including hypoplastic left heart syndrome (HLHS) and single ventricle physiology. The global burden of CHD continues to grow, with an estimated 11.9 million individuals living with CHD worldwide as of 2017, representing an 18.7% increase from 1990 [3].

### Clinical burden of CHD

The clinical landscape of CHD has undergone dramatic transformation over the past five decades. What was once a uniformly fatal condition has evolved into a chronic disease requiring lifelong management, thanks to remarkable advances in paediatric cardiac surgery, interventional catheterization, and intensive care. However, improved survival has unveiled new challenges across the lifespan. Neonates with critical CHD require immediate intervention, often within the first 24–48 h of life, with prostaglandin E infusion and emergency surgery serving as life-saving interventions [4]. In addition, the economic burden of CHD is substantial and growing. Beyond direct medical costs, families face significant indirect costs including lost productivity, travel for specialized care, and long-term disability support. The psychosocial impact is equally profound, with increased rates of depression, anxiety, and neurodevelopmental disorders affecting quality of life across all age groups [5, 6].

### Molecular complexity of CHD

The pathogenesis of CHD arises from disruptions in tightly regulated cardiac developmental programs. Normal heart formation depends on precise coordination of signalling pathways, including Wnt/β-catenin for progenitor specification, NOTCH for chamber and valve formation, and BMP/TGF-β for extracellular matrix (ECM) organization and cushion development [7, 8]. Even subtle disturbances in the timing or location of these pathways can lead to structural defects. Heart development is also tightly regulated by a diverse set of heart stem/progenitor cells in a spatiotemporal manner at the earliest embryonic stage [9, 10]. Consequently, dysfunction of these heart stem/progenitor cells and/or their paracrine molecular cues in developing hearts likely causes the onset of CHD.

Genetic factors contribute substantially to CHD risk but are heterogeneous. De novo mutations occur in 8–10% of cases, enriched in genes for chromatin modifiers, transcription factors, and signalling molecules [11, 12]. Most CHD cases, however, lack a clear monogenic cause, suggesting polygenic and environmental contributions. Genome-wide studies have identified > 100 risk loci, though they explain only a fraction of heritability [13].

At the cellular level, CHD involves dysfunction across multiple cardiac lineages. Cardiomyocytes (CMs) may show impaired proliferation or metabolic maturation; endocardial cells display abnormal endothelial-to-mesenchymal transition (EndMT); and cardiac neural crest cells (NCCs) exhibit migratory or differentiation defects underlying outflow tract malformations [14–16]. Understanding these lineage-specific disruptions requires technologies capable of resolving cellular heterogeneity, such as single-cell omics, to map the molecular architecture of CHD.

### Single-cell technologies transform CHD research

Single-cell RNA sequencing (scRNA-seq) has revolutionized the study of CHD by revealing cell type–specific transcriptional programs, rare populations, and transitional states that bulk RNA sequencing cannot resolve [17]. This technology is especially valuable for cardiac development and disease where cellular heterogeneity and dynamic processes shape pathogenesis.

Advances in single-cell analytic methods have been rapid. Early low-throughput approaches analysed only dozens of cells, whereas current droplet- (10x Genomics, Drop-seq) and plate-based (Smart-seq, CEL-seq) platforms can profile millions. The advent of single-nucleus RNA sequencing (snRNA-seq) has been crucial for cardiac tissue, enabling analysis of adult CMs that are otherwise difficult to isolate. Newer modalities, spatial transcriptomics and multimodal omics, now integrate gene expression with chromatin accessibility and protein abundance [18] to reveal the novel developmental trajectories relevant to CHD, especially conotruncal heart malformation (CTM) such as tetralogy of Fallot (TOF) [10] or to generate comprehensive atlases of cardiac development and CHD, identifying cell type–specific defects and transcriptional programs underlying disease [19]. Furthermore, integration with patient-derived induced pluripotent stem cell (iPSC) models allows functional validation and therapeutic screening at single-cell resolution [20].

This review synthesizes recent single-cell omics research in human cardiogenesis and CHD, cataloguing technologies and findings across subtypes to identify convergent mechanisms and therapeutic opportunities. We further present an in-depth reanalysis of the Hill et al. (2022) dataset [15] using advanced computational tools to map intercellular communication and prioritize signalling pathways with translational potential.

## Single-cell landscape in CHD research

### Overview of cardiac single-cell omics studies

Since 2019, single-cell technologies have transformed CHD research, spanning early cardiac development to adult disease. Our review identified 17 major cardiac single-cell omics studies (2019–2025) that collectively advanced understanding of human cardiogenesis and CHD pathogenesis (Table 1). These works employed diverse platforms and sample types, uncovering cell type-specific mechanisms across multiple CHD subtypes.

Early studies using plate-based methods (e.g., Smart-seq2) enabled deep profiling of hundreds of cells, while droplet-based technologies (10x Genomics, Drop-seq) expanded coverage to tens of thousands, revealing rare and transitional populations [10, 15]. The advent of spatial transcriptomics has revealed how gene expression maps onto tissue architecture, and multimodal approaches have begun linking transcriptomic and chromatin features [21].

For cardiac tissue, single-nucleus RNA sequencing (snRNA-seq) became the preferred strategy because mature CMs are difficult to isolate intact. The Hill et al. (2022) study exemplifies this approach, profiling over 157,000 nuclei from paediatric CHD hearts and establishing a benchmark dataset for disease-specific single-cell analysis.

### Cardiac development at single-cell resolution

The heart develops through a precisely orchestrated sequence of morphogenetic events, beginning with the specification of cardiac progenitors and culminating in the formation of a four-chambered organ with specialized conduction system, valves, and coronary vasculature. Understanding normal cardiac development at single-cell resolution provides the essential foundation for interpreting disease mechanisms in CHD.

**Table 1:** Summary of major single-cell omics studies in human cardiogenesis and CHD (2019–2025)

Author (year)	Stage of analysed samples	Modalities	Key insights
Asp et al. (2019) [21]	Embryonic hearts (4.5–9 wks)	scRNA-seq + spatial transcriptomics	Organ-wide gene-expression atlas mapped to anatomical regions
Sahara et al. (2019) [10]	Embryonic heart (~ 4–10 wks)	scRNA-seq + bulk RNA-seq	Identified LGR5⁺ cono-ventricular progenitors in outflow tract
Cui et al. (2019) [19]	Foetal hearts (5–25 wks)	scRNA-seq	Chamber-specific cell types & signalling pathways during development
Cao et al. (2020) [22]	Foetal organs (including heart)	scRNA-seq	Pan-organ developmental gene atlas including heart
Miao et al. (2020) [16]	Foetal endocardial cells (HLHS)	scRNA-seq	Endocardial defects in HLHS; developmental disruptions in CHD
Litviňuková et al. (2020) [23]	Adult hearts (multiple donors)	snRNA-seq + Visium ST	Cell type composition across cardiac chambers; sex differences
Krane et al. (2021) [24]	HLHS foetal heart tissues	scRNA-seq	Progenitor lineage defects leading to HLHS
Sim et al. (2021) [25]	Foetal to adult hearts	snRNA-seq + ATAC-seq + functional assays	Sex-specific role of progesterone receptor in cardiomyocyte maturation
Ameen et al. (2022) [26]	Foetal hearts (6–22 wks)	Multiome (snRNA-seq + snATAC-seq)	Chromatin & transcriptional profiles; cell fate regulation
Hill et al. (2022) [15]	Paediatric CHD patients’ hearts (0–17 yrs)	scRNA-seq + bulk ATAC-seq	Immune cell chromatin accessibility linked to CHD subtypes
Farah et al. (2023) [27]	Foetal hearts (9–16 wks)	scRNA-seq + MERFISH ST	Spatiotemporal atlas; signalling in morphogenesis
Queen et al. (2023) [28]	CHD vs. normal foetal hearts	snRNA-seq	Transcriptional dysregulation in CHD; TF networks
Kanemaru et al. (2023) [29]	Adult hearts (25 donors)	snRNA-seq + Visium ST	Adult heart atlas; conduction & regulatory maps
Cranley et al. (2023) [30]	Embryonic hearts (4.5–7 wks)	scRNA-seq	Temporal cardiac morphogenesis trajectories
Hou et al. (2024) [31]	Foetal hearts (8–17 wks)	scRNA-seq	Maturation signatures & atrial-ventricular divergence
He et al. (2025) [32]	Foetal heart chambers	Visium ST	Spatial TF expression domains in atria vs. ventricles
De Brono et al. (2025) [33]	Foetal & neonatal hearts	scRNA-seq, Visium ST, immunostaining	Neural crest & epicardial lineages in 3D spatial context

*ATAC-seq, assay for transposase-accessible chromatin sequencing; ST, spatial transcriptomics; TF, transcription factor

#### Embryonic heart atlas studies

Asp et al. [21] generated the first spatiotemporal atlas of human heart development, analysing three developmental stages during the first trimester. This landmark study combined single-cell RNA sequencing with spatial transcriptomics to reveal how gene expression patterns relate to anatomical structures. Key findings included the identification of chamber-specific gene expression programs, with *NKX2.5* showing dynamic expression patterns that correlate with ventricular specification and *TBX5* exhibiting left-sided enrichment consistent with its role in Holt-Oram syndrome. Subsequently, the discovery of LGR5^+^ cono-ventricular progenitors revealed previously unrecognized heterogeneity within cardiac stem/progenitor populations that are classically represented by MESP1^+^ cardiac mesodermal precursors, and first (NKX2.5^+^TBX5^+^) and second (ISL1^+^) heart field progenitors [10]. These LGR5^+^ cells showed unique transcriptional profiles and developmental trajectories, suggesting that disruption of specific progenitor subpopulations could underlie chamber-specific defects in CHD such as CTM. Specifically, the LGR5^+^ population emerges in the proximal outflow tract at 4–5 weeks of foetal age and drives cardiac alignment through expansion of ISL1^+^TNNT2^+^ intermediates [10]. Transcriptionally, these cells were enriched for Wnt/β-catenin signalling components, and functional studies showed that *LGR5* knockdown impaired CM differentiation while promoting endothelial fate, indicating *LGR5* acts as a lineage commitment switch [10, 34]. Single-cell analyses of murine cardiogenesis have further demonstrated that loss of specific progenitor subpopulations can produce chamber-specific defects [14], supporting the concept that disruption of the LGR5^+^ module could specifically impair outflow tract morphogenesis and contribute to CTM.

Cui et al. (2019) [19] focused on human foetal heart development, profiling over 4,000 cardiac cells across multiple developmental stages and identified four major cell types, CM, cardiac fibroblast (CF), endothelial cell (EC), and smooth muscle cell (SMC), and characterized their developmental trajectories. Importantly, they showed that many CHD-associated genes exhibit highly restricted temporal expression windows, suggesting critical periods for therapeutic intervention.

#### Key developmental insights

Heart field specification, the earliest stage of cardiac development, segregates progenitors into the first (FHF) and second heart fields (SHF). Single-cell trajectory analyses reveal that this occurs earlier than previously thought and involves distinct transcriptional programs. Murine lineage tracing studies previously revealed that FHF progenitors (TBX5^+^, HCN4^+^) form the left ventricle and atria, whereas SHF progenitors (ISL1^+^, FGF10^+^) give rise to the right ventricle, outflow tract, and parts of the atria [35]. Further, recent scRNA-seq studies using human embryonic or foetal hearts have identified intermediate progenitors, that transiently emerge and co-express both immature mesodermal and cardiac genes, indicating critical decision points [10, 19]. Chamber formation proceeds through coordinated proliferation and differentiation of cardiac progenitors. Transcription factors *HAND1*, *HAND2*, and *IRX4* display chamber-restricted expression that is often disrupted in CHD [36, 37].

During valve development, single-cell analysis of endocardial cushions has defined multiple transitional states in EndMT, marked by progressive loss of endothelial genes (*CDH5*, *PECAM1*) and gain of mesenchymal markers (*VIM*, *SNAI1*) [38]. Dynamic activation of NOTCH, BMP, and WNT pathways regulates this process of valve formation [39].

Finally, cardiac NCCs have been mapped at single-cell resolution, showing essential contributions to outflow tract septation and great vessel patterning [40]. Trajectory analyses reveal transitions guiding neural crest toward smooth muscle and other lineages [40, 41], and their disruption underlies conotruncal defects, including those associated with 22q11.2 deletion syndrome [42].

### CHD-specific single-cell findings

The application of single-cell technologies to CHD patient samples and models has revealed disease-specific cellular and molecular mechanisms that were invisible to bulk analysis approaches. These studies have identified convergent and divergent mechanisms across CHD subtypes, providing insights into both shared vulnerabilities and lesion-specific defects.

#### Hypoplastic left heart syndrome

HLHS represents one of the most severe forms of CHD, characterized by underdevelopment of left-sided cardiac structures. Single-cell studies have revealed multiple cellular mechanisms contributing to HLHS pathogenesis, challenging the traditional view of HLHS as simply a “small left ventricle” condition.

Hill et al. [15] analysed 157,273 nuclei from HLHS patients and controls, revealing profound alterations across multiple cell types. HLHS CM showed insulin resistance, metabolic dysfunction, and increased *FOXO1* target gene expression, suggesting chronic metabolic stress. Elevated *CRIM1* expression potentially impaired proliferation and maturation, indicating metabolic interventions as potential therapeutic approaches. HLHS CF demonstrated high YAP activity and low Hippo signalling, with increased ECM genes and pro-fibrotic factors contributing to myocardial fibrosis. Trajectory analysis revealed HLHS CF arrested in intermediate activation states, identifying *YAP* as a potential therapeutic target.

Miao et al. (2020) [16] identified intrinsic endocardial defects in HLHS using patient-derived iPSCs. Single-cell analysis revealed impaired NOTCH signalling, reduced valve development genes, and failed EndMT. These endocardial defects had non-cell-autonomous effects on CM differentiation, contributing to myocardial hypoplasia. HLHS also showed immunodeficiency with reduced macrophage infiltration and altered cytokine signalling, potentially explaining increased infection susceptibility. Resident macrophages exhibited reduced tissue-protective factors and inflammatory signatures [15]. Xu et al. [43] further elucidated the mechanism of early heart failure in HLHS using iPSC-derived CMs from 10 HLHS patients dichotomized by cardiac outcome. Single-cell transcriptomics confirmed that early heart failure was associated with mitochondrial dysfunction and endoplasmic reticulum (ER) stress, whereas patients with transplant-free survival more than 5 years showed normal respiration with elevated antioxidant response. iPSC-CMs of early heart failure group exhibited increased apoptosis, mitochondrial respiration defects, and redox stress from abnormal mitochondrial permeability transition pore (mPTP) opening with failed antioxidant response, as well as reduced proliferation, CM maturation defects (lower *MYH6*/*MYH7* ratio), myofibrillar disarray, and impaired contractility [43]. Importantly, these mitochondrial and ER stress defects were rescued by sildenafil or tauroursodeoxycholic acid treatment, suggesting these agents as potential therapeutic avenues for HLHS-associated heart failure [43]. Krane et al. (2021) demonstrated that HLHS results from sequential developmental defects, with iPSC studies showing delayed cardiac gene activation and persistent progenitor markers [24].

#### Conotruncal defects

Conotruncal defects encompass a spectrum of outflow tract abnormalities, including TOF, truncus arteriosus, double outlet right ventricle (DORV), and transposition of the great arteries (TGA), which share common developmental origins involving SHF. TOF, the most common cyanotic CHD, is characterized by four anatomical features: VSD, pulmonary stenosis, overriding aorta, and right ventricular hypertrophy [44]. Single-cell studies have revealed that these defects arise from disruptions in SHF development and neural crest migration [40]. iPSC-derived CMs from TOF patients showed extensive transcriptional dysregulation with 250 differentially expressed genes (DEGs) [45]. Key dysregulated transcription factors included *NR2F2* (atrial identity) and *HEY2* (NOTCH target for ventricular septation), with aberrant atrial gene expression in ventricular-like cells suggesting chamber specification defects [7, 46]. Genomic studies identified enrichment of de novo mutations in histone modifiers and chromatin regulators, indicating epigenetic dysregulation [47]. Copy number variants at chromosome 22q11.2, found in ~ 15% of TOF cases, highlight *TBX1* dosage importance in outflow tract development [48].

Spatial transcriptomics revealed disrupted tissue organization with altered gene expression gradients. Outflow tract markers expanded into right ventricular regions, consistent with overriding aorta phenotype, suggesting tissue-level organizational defects beyond cell-autonomous abnormalities [27]. Neural crest analysis showed migration defects and failed transition from multipotent progenitors to SMCs, remaining in intermediate states. *GATA6* emerged as a critical regulator, with mutations causing persistent truncus arteriosus [49–51]. Other studies revealed reduced proliferation and premature differentiation of SHF progenitors in TOF patients, creating smaller progenitor pools. Altered signalling responses included reduced WNT responsiveness and enhanced BMP signalling, disrupting proliferation-differentiation balance and contributing to hypoplastic right ventricular outflow tract [52, 53]. Metabolic profiling identified shifts toward glycolysis with reduced oxidative phosphorylation, associated with altered mitochondrial gene expression and reduced peroxisome proliferator-activated receptor gamma coactivator 1 alpha (*PGC1α*), a master transcriptional coactivator of mitochondrial biogenesis and oxidative metabolism in CMs [54]. These metabolic changes may contribute to right ventricular hypertrophy and dysfunction during hemodynamic stress [55, 56].

DORV and TGA involve disruption of left-right signalling pathways. DORV pathogenesis involves *Pitx2*-mediated asymmetric morphogenesis, while TGA has been linked to laterality defects with enrichment of ciliary genes essential for left-right axis determination [57].

#### Other CHD subtypes

Single ventricle disease, a severe CHD characterized by functional absence of one ventricle, revealed the most extensive transcriptional perturbations among CHD subtypes, with 919 DEGs affecting sarcomere organization, calcium handling, and metabolism alongside chronic cellular stress with interferon signalling and NF-κB activation [45].

Hypoplastic right heart syndrome (HRHS) displays distinct molecular signatures from HLHS, with specific defects in right ventricular CM maturation, persistent embryonic sarcomeric isoforms, and reduced *HAND2* expression [58, 59].

VSDs involve multiple cell types with muscular septum formation requiring coordinated proliferation and differentiation signals regulated by dosage-sensitive *TBX1* [58, 60]. Atrioventricular septal defects (AVSDs), common in Down syndrome, showed disrupted endocardial cushion development, with chromosome 21 genes *DSCAM* and *COL6A1*/*2* showing dose-dependent effects on cushion fusion [61, 62].

Concurrently, the development of sophisticated cardiac tissue engineering approaches has revolutionized CHD modelling capabilities. Advanced platforms including vascularized cardiac organoids and engineered heart tissues offer unprecedented opportunities for studying complex CHD subtypes [17, 18, 63, 64]. These three-dimensional models more accurately recapitulate the cellular interactions and mechanical forces characteristic of developing hearts, providing enhanced systems for elucidating disease mechanisms and evaluating therapeutic interventions [65, 66].

Despite advances in single-cell CHD research, disease heterogeneity remains incompletely understood. Large-scale datasets enable identification of shared pathological pathways across CHD subtypes. We reanalysed the Hill et al. dataset [15] to systematically examine intercellular communication networks across single nuclei from multiple CHD conditions, revealing novel insights into disease-specific molecular alterations and potential therapeutic targets.

## Re-analysis of Hill et al. dataset: intercellular communication networks in CHD

### Dataset overview

The Hill et al. (2022) dataset (https://www.ncbi.nlm.nih.gov/geo/query/acc.cgi?acc=GSE203275) is the most comprehensive single-cell study of CHD to date, profiling 157,273 nuclei from paediatric patients across six diagnostic groups (Table 2). We reanalysed this dataset using advanced computational methods with a focus on ligand-receptor interactions [67–69] to uncover potential therapeutic targets for precision medicine.

### Data processing

The data analysis pipeline flowchart is shown in Supplementary Fig. 1.

#### Data quality control and preprocessing

Quality control included assessment of mitochondrial (^MT-) and ribosomal (^RP[SL]) gene percentages using PercentageFeatureSet. To ensure data quality, cells with excessively high mitochondrial content (> 20%) or abnormal feature counts (< 200 or > 6,000 detected genes) were excluded. Data were normalized with the LogNormalize method (scale factor = 10,000), and 2,000 highly variable genes were identified via variance-stabilizing transformation. Principal component analysis (PCA) was performed on scaled data using these features, and the top 30 principal components were used for Uniform Manifold Approximation and Projection (UMAP) dimensionality reduction to generate two-dimensional embeddings for visualization. Cell type identities were validated by examining expression of established marker genes across clusters.

#### Differential expression and pathway analysis

Differential gene expression was assessed using the Wilcoxon rank-sum test via Seurat’s FindMarkers function, with significance thresholds of adjusted *P* < 0.05. Analyses compared each disease condition against healthy donor controls, requiring expression in ≥ 10% of cells (min.pct = 0.1). For cell type–specific comparisons, datasets were subset by cell type prior to testing. Expression heatmaps were generated using hierarchical clustering (Euclidean distance, complete linkage) and z-score normalization across genes. The top 200 DEGs were used for overall condition comparisons, while top 50 DEGs per cell type (ranked by adjusted *P*-value) were selected for focused analyses.

#### Intercellular communication analysis

Intercellular communication was evaluated by quantifying ligand–receptor interactions relevant to cardiac signalling networks [67–69]. Communication strength between cell types was quantified using two metrics:

- Crosstalk Potential = mean ligand expression (sender) × mean receptor expression (receiver).
- Crosstalk Score = √(percentage of ligand-expressing cells × percentage of receptor-expressing cells).

Mean values were derived from normalized RNA counts, with a minimum of 10 cells per type per condition required for analysis. Four directional interactions were examined: CF to CM, CM to EC, EC to CM, and CM to CF. Cardiac NCCs were not included as they were undetectable in this postnatal dataset, consistent with their transient presence during early embryonic development. Epicardial cells were present at very low abundance and restricted primarily to healthy donor (Donor) and infant HLHS (IF_HLHS) groups. These cells were therefore analysed separately alongside CM, CF, and EC. Disease-specific alterations were expressed as fold changes relative to healthy donor controls for individual pairs and functional categories.

Network topology visualization [70] depicted cell types as nodes and interaction strengths as weighted edges, ranked by mean crosstalk potential. All analyses and figures were generated in R using *ggplot2* and custom scripts for network diagram construction.

#### Computational environment

All analyses were performed in R v4.2.0 on the SHIROKANE supercomputer (Human Genome Centre, Institute of Medical Science, University of Tokyo). Core packages included *Seurat* (v4.0) for single-cell analysis, dplyr (v1.0.9) for data handling, ggplot2 (v3.4.0) for visualization, and ComplexHeatmap (v2.12.0) and pheatmap (v1.0.12) for heatmap generation. The analysis code is available from the authors upon request.

### Key findings

#### Dataset characterization and quality assessment

**Table 2:** Clinical characteristics and cell count summary of the Hill et al. (2022) CHD dataset

Diagnosis	Number of samples	Age range (years)	Gender (Male : Female)	Number of cells
Healthy donor (Donor)	4	3–11	1 : 3	25,847
Tetralogy of Fallot (TOF)	3	0	1 : 2	31,562
Neonatal hypoplastic left heart syndrome (Neo_HLHS)	1	0	1 : 0	28,951
Infant hypoplastic left heart syndrome (IF_HLHS)	2	3–4	2 : 0	22,184
Dilated cardiomyopathy (DCM)	2	16–17	2 : 0	24,729
Hypertrophic cardiomyopathy (HCM)	1	11	0 : 1	24,000

**Fig. 1:**
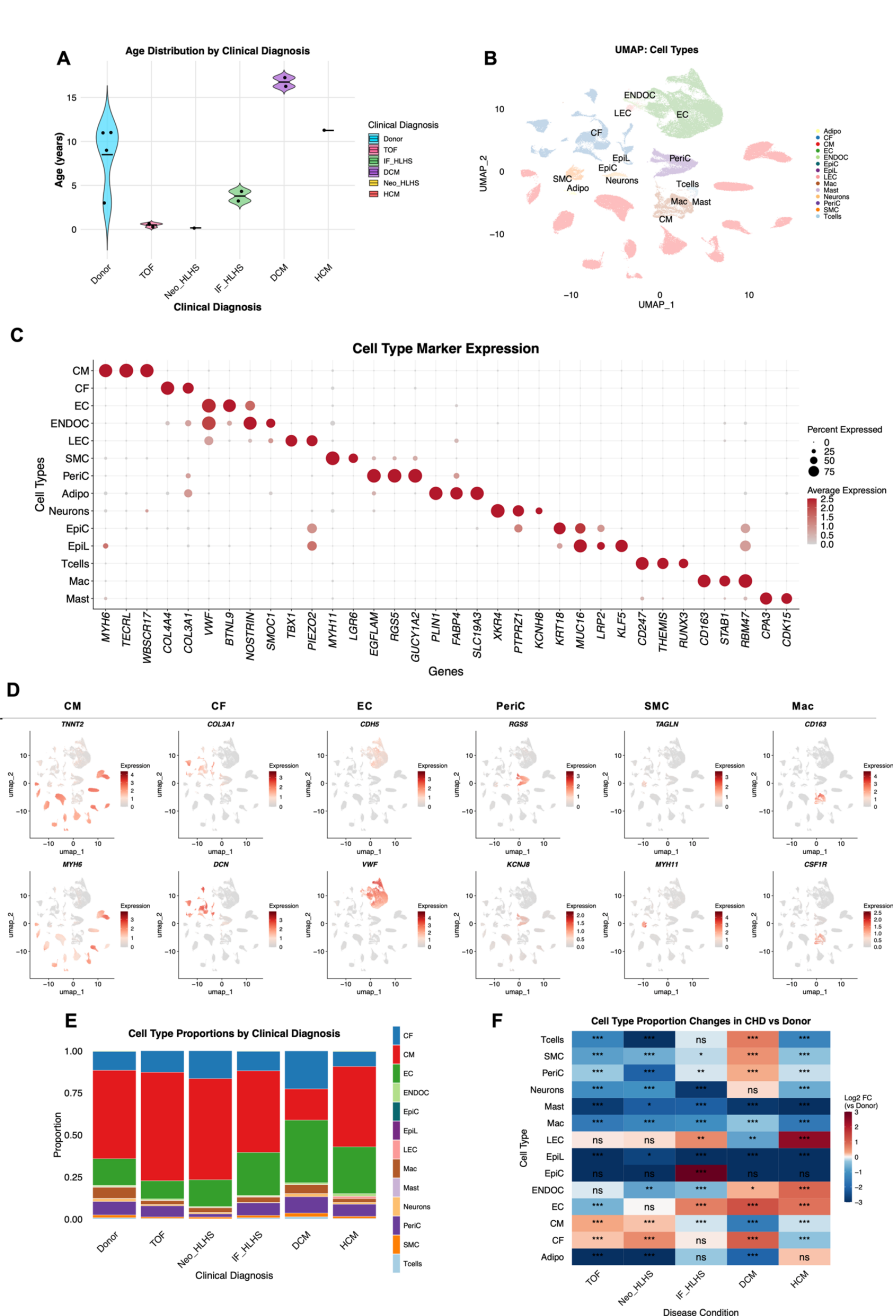
Dataset characterization and cell type identification. **A** Age distribution across clinical conditions. Violin plots showing age distribution of patients by clinical diagnosis. Individual patients shown as coloured dots. Black horizontal line indicates mean age. Donor (*n* = 4): healthy donor hearts; TOF (*n* = 3): Tetralogy of Fallot; Neo_HLHS (*n* = 1): neonatal hypoplastic left heart syndrome; IF_HLHS (*n* = 2): infant hypoplastic left heart syndrome; DCM (*n* = 2): dilated cardiomyopathy; HCM (*n* = 1): hypertrophic cardiomyopathy. Ages in years. **B** UMAP visualization by cell type. UMAP embedding of all cells coloured by major cardiac cell type, showing distinct cellular clusters. Adipo (adipocyte), CF (cardiac fibroblast), CM (cardiomyocyte), EC (endothelial cell), ENDOC (endocardial cell), EpiC (epicardial cell), EpiL (epithelial-like cell), LEC (lymphatic endothelial cell), Mac (macrophage), Mast (mast cell), Neurons, PeriC (pericyte), SMC (smooth muscle cell), and Tcells (T cells). **C** Cell type marker expression. Dot plot showing expression of canonical marker genes across cardiac cell types. Dot size represents the percentage of cells expressing each gene; colour intensity indicates average expression level (blue = low, red = high). **D** Feature plots of marker gene expression. UMAP feature plots displaying spatial expression patterns of representative marker genes. Expression intensity shown on gray-to-red colour scale. **E** Cell type composition by clinical conditions. Stacked bar plots showing the proportional representation of major cardiac cell types across conditions. **F** Cell type proportion changes across CHD conditions. Heatmap showing log_2_ fold-change in cell type proportions for each CHD condition versus healthy donor control (Donor). Statistical significance was assessed by Fisher’s exact test with Benjamini-Hochberg (BH) correction for each pairwise comparison (each CHD condition vs. Donor). Rows represent the 14 major cardiac cell types; columns represent the five CHD conditions. Colour scale indicates the magnitude and direction of proportion change (red = increased proportion; blue = decreased relative to Donor; capped at ±3 log_2_FC. Significance annotations: ns, not significant; *, FDR < 0.05; **, FDR < 0.01; ***, FDR < 0.001

Patients ranged from 0.2 to 17.3 years of age (Fig. 1 A; Table 2). UMAP visualization confirmed successful integration across conditions while preserving biological variation (Fig. 1 B, Supplementary Fig. 2 A). The dataset comprised 14 major cardiac cell types: CM, CF, EC, SMC, pericyte (PeriC), adipocyte (Adipo), epicardial cell (EpiC), epithelial-like cell (EpiL), endocardial cell (ENDOC), mast cell (Mast), macrophage (Mac), T-cells (Tcells), lymphatic endothelial cell (LEC), and neurons. Cell type clusters showed clear separation with minimal batch effects, validating technical quality for downstream analysis. Cell type identities were validated by established marker genes across clusters (Fig. 1 C), with feature plots demonstrating spatial organization of expression patterns across UMAP embedding (Fig. 1 D).

Cellular composition varied markedly across the six diagnostic groups (Fig. 1 E, Supplementary Table 1). To further characterize the directionality and magnitude of these compositional changes relative to healthy donors, pairwise log_2_ fold changes (log_2_FC) were shown in Fig. 1F. CM was significantly increased in TOF (64.5%) and neonatal HLHS (Neo_HLHS) (60.1%) but markedly reduced in dilated cardiomyopathy (DCM) (18.5%) (FDR < 0.001, Supplementary Table 1). CF was significantly elevated in DCM (22.6%), Neo_HLHS (16.6%), and TOF (12.8%) (FDR < 0.001, Supplementary Table 1), but unchanged in IF_HLHS. EC was significantly elevated in IF_HLHS, DCM and hypertrophic cardiomyopathy (HCM) (FDR < 0.001). Propeller testing [71] across all CHD conditions versus healthy donor controls identified CM, Tcells, Mast, Mac, and LEC as populations showing statistically significant cell-proportion changes (FDR < 0.05), while EC was approaching significance (FDR = 0.059) (Supplementary Fig. 2B).

#### Divergent cell-type-specific transcriptional responses across CHD subtypes

**Fig. 2:**
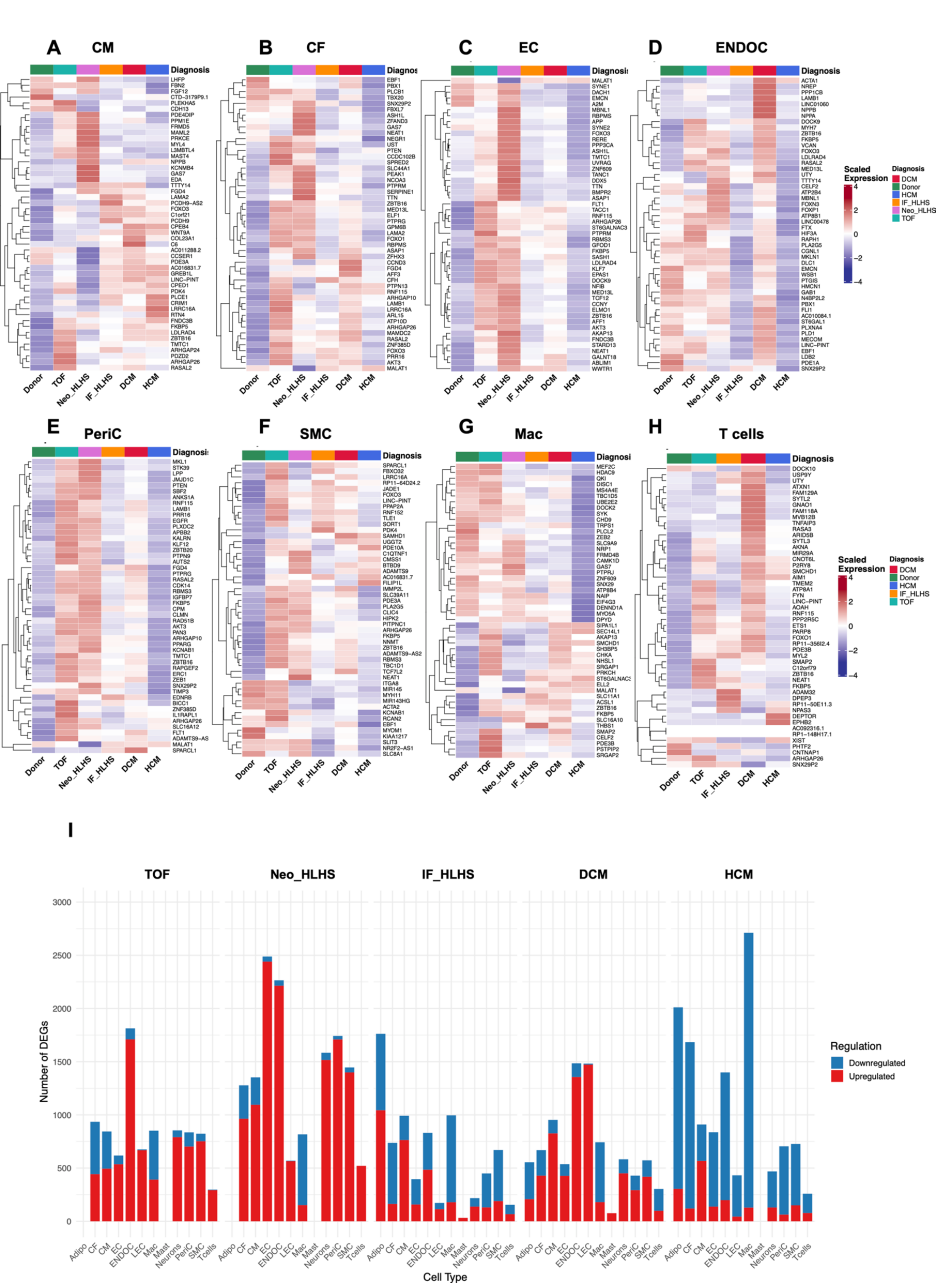
Cell type-specific differential gene expression across cardiac disease conditions. **A**–**H** Heatmaps showing the top 50 cell type-specific differentially expressed genes (DEGs). **A** Top 50 DEGs in cardiomyocyte (CM). **B** Top 50 DEGs in cardiac fibroblast (CF). **C** Top 50 DEGs in endothelial cell (EC). **D** Top 50 DEGs in endocardial cell (ENDOC). **E** Top 50 DEGs in pericyte (PeriC). **F** Top 50 DEGs in smooth muscle cell (SMC). **G** Top 50 DEGs in macrophage (Mac). **H** Top 50 DEGs in T cells (Tcells). Expression values are z-score normalized (red = high expression, blue = low expression) with hierarchical clustering applied to genes. Colour bars indicate disease conditions (Donor, TOF, Neo_HLHS, IF_HLHS, DCM, HCM). **I** Cell type-stratified differential expression summary. Bar plot showing the number of DEGs by cell type across all disease conditions. Red bars represent upregulated genes; blue bars represent downregulated genes

Comprehensive differential expression analysis revealed extensive transcriptional remodelling across all disease conditions, with each exhibiting unique cell type-specific signatures (Fig. 2A–I, Supplementary Tables 2–3). The magnitude of transcriptional disruption varied substantially: Neo_HLHS showed the most profound alterations (6,822 DEGs; 96.4% of 7,077 genes tested), followed by TOF (4,764 DEGs; 96.8% of 4,924 genes), while IF_HLHS, HCM, and DCM displayed comparatively moderate but still substantial changes (3,778–4,948 DEGs; >90% of tested genes) (Supplementary Table 2A-C).

##### Vascular-endothelial and endocardial activation define congenital heart malformations

EC showed striking disease-specific patterns. Neo_HLHS EC exhibited near-complete transcriptional activation (2,609 DEGs, 98.2% upregulated), with prominent induction of angiogenic regulators (*HIF1A*, *ANGPT2*), endothelial identity genes (*ENG*, *TIE1*), vascular tone mediators (*EDN1*), and pro-inflammatory adhesion molecules (*SELE*, *CCL2*), alongside EndMT markers (Fig. 2C, Supplementary Table 3). In contrast, HCM EC was predominantly downregulated (812 DEGs, 17.1% upregulated), with loss of EC identity markers (*TIE1*, *TEK*), and suppression of *VCAM1* and *CXCL12*, suggesting impaired endothelial function. TOF EC showed relatively similar patterns to those in Neo_HLHS, while IF_HLHS EC displayed intermediate activation patterns (Fig. 2C, Supplementary Table 3).

This vascular remodelling appeared to extend to periC and SMC. In Neo_HLHS, periC strongly upregulated *FGD4*, *PTEN*, *RASAL2*, and *FLT1* (1,824 DEGs, 98.1% upregulated) (Fig. 2E, Supplementary Table 2 C-3). SMC in TOF displayed extensive transcriptional activation (818 DEGs, 92.1% upregulated) (Fig. 2 F, Supplementary Table 2 C-3). Collectively, EC, ENDOC, and PeriC exhibited maximal transcriptional remodelling in TOF and Neo_HLHS (Fig. 2I).

ENDOC showed prominent developmental programs in TOF and Neo_HLHS (1,610 and 2,038 DEGs, 94.7% and 96.9% upregulated respectively. In Neo_HLHS, ENDOC activated basement membrane genes (*LAMC2*, *HSPG2*), EndMT markers (*ZEB1*, *FN1*), and inflammatory mediators (*SELE*). In TOF, ENDOC upregulated valve development genes (*BMPR2*, *LAMB1*) (Fig. 2D, I, Supplementary Table 2 C-3). In contrast, ENDOC of HCM clustered distinctly with widespread suppression of EndMT markers and ECM genes, revealing fundamentally divergent cellular strategies across different cardiac disease aetiologies (Supplementary Table 3).

##### Cardiomyopathies display divergent myocardial–stromal programs

Despite shared classification as cardiomyopathies, DCM and HCM exhibited fundamentally distinct CM signatures. DCM drove a predominantly upregulated response (951 DEGs, 86.9% upregulated) with dual activation of cardiac stress markers (*NPPA*, *NPPB*) and metabolic reprogramming (*PDK4*, *HK2*), alongside suppression of sarcomeric genes (*MYL7*, *ACTC1*) and the mechano-stress sensor *ANKRD1*. HCM showed a mixed response (903 DEGs, 62.9% upregulated) with shared stress marker induction but unique Ca²⁺ dysregulation — activating *CALM1* while suppressing *PLN* and *PKM* and distinct sarcomeric remodelling via *MYH6* (Fig. 2A, I; Supplementary Table 2 C).

HCM displayed profound transcriptional repression across stromal lineages. CF showed marked downregulation including mechano-sensing genes (PLCB1), ECM components *(TIMP2*,* COL6A1)*, and the TGF-β mediator *SMAD3* (1,685 DEGs, only 7.0% upregulated) (Fig. 2B, I and Supplementary Table 2 C-3). PeriC and SMC similarly repressed contractility genes (*ACTA2*) (Fig. 2E–F).

In sharp contrast, CF of DCM retained expression of contractility markers and activated ECM remodelling regulators (*SERPINE1*, *LAMB1*, *FNDC3B*) (650 DEGs, 66.6% upregulated) (Fig. 2B, I). This striking context-dependent plasticity in CF, which was strongly repressed in HCM (92.9% downregulated), moderately activated in DCM (65.6% upregulated) and intermediate in IF_HLHS (20.1% upregulated), exemplifies how various CHD conditions manifest through opposite cellular mechanisms. In DCM, EC and LEC emerged as dominant responders, while stromal cells mounted adaptive upregulation (Fig. 2I, Supplementary Table 2 A, C).

##### Immune compartments show disease-specific polarization

Macrophages displayed condition-specific polarization patterns (Fig. 2G, I, Supplementary Table 2 C-3). TOF Mac showed mixed activation (948DEGs, 43.5% upregulated), with concurrent induction of M1 markers (*HLA-DRA*, *CD86*) and M2 markers (MRC, CD163), alongside efferocytosis receptors (*AXL*, *MERTK*). Neo_HLHS and DCM Mac was predominantly downregulated (14.6% and 22.3% upregulated respectively), with *IL1B* induction among the few induced genes in Neo_HLHS. HCM Mac showed the most profound suppression across all conditions (2,903 DEGs, only 4% upregulated), with broad loss of tissue remodelling (*LGMN*, *GPNMB*) and phagocytic capacity (*MARCO*) (Fig. 2G, I, Supplementary Table 2 C-3).

T cells revealed a conserved stress-response signature across all diseases centred on *ZBTB16*, *NEAT1*, and *FKBP5* (Fig. 2H, I, Supplementary Table 2 C-3).

#### Intercellular communication network remodelling in CHD

**Fig. 3:**
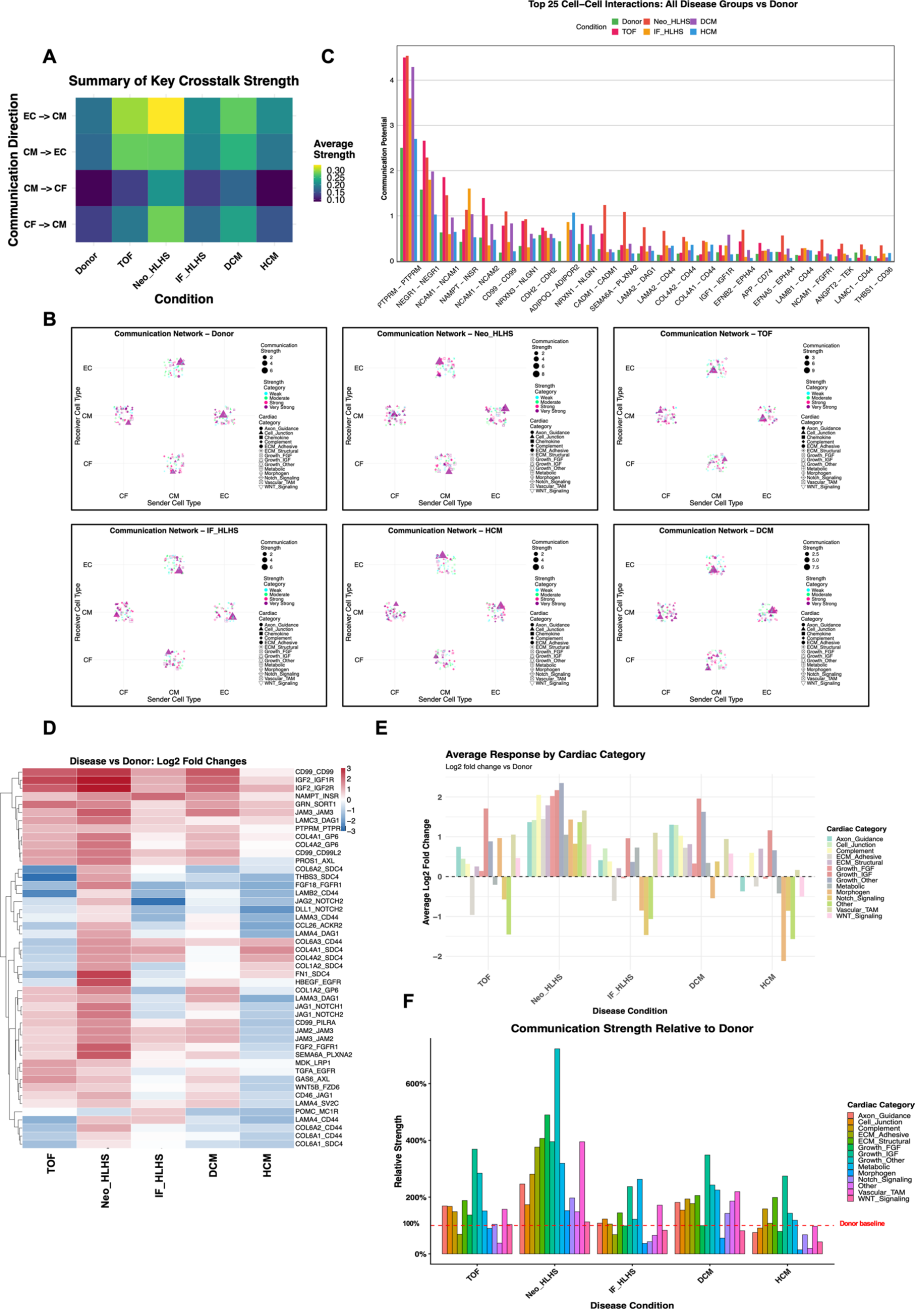
Intercellular communication network reorganization in cardiac disease. (**A**) Cell type-specific crosstalk strength heatmap. Heatmap showing average crosstalk potential between cardiac cell type pairs (CF to CM, CM to EC, EC to CM, CM to CF) across conditions (Donor, TOF, Neo_HLHS, IF_HLHS, DCM, HCM). Colour intensity represents communication strength (darker blue = weaker, green = intermediate, and yellow = stronger). **B** Network topology by respective conditions. Network diagrams for each of six conditions (Donor, Neo_HLHS, TOF, IF_HLHS, HCM, DCM) showing sender-receiver relationships between EC and CM, and between CF and CM. Node size and position indicate cell type; point size and colour represent communication strength and functional category. **C** Top 25 strongest ligand-receptor interactions. Bar plot ranking the 25 most robust cardiac ligand-receptor pairs by overall communication potential across all conditions. Bars are color-coded by clinical conditions (Donor, TOF, Neo_HLHS, IF_HLHS, DCM, HCM). **D** Interaction-specific fold changes across conditions. Clustered heatmap showing log2 fold changes of cardiac-specific ligand-receptor interactions versus Donor controls. Rows represent individual interactions, columns represent conditions (TOF, Neo_HLHS, IF_HLHS, DCM, HCM). Colour scale: blue (downregulated, −3 log2FC) to red (upregulated, + 3 log_2_FC). **E** Average response by cardiac category. Bar plot showing average log_2_fold change versus Donor controls for each functional pathway category across disease conditions (TOF, Neo_HLHS, IF_HLHS, DCM, HCM). Each bar represents a functional category as indicated by colour legend. The dashed line at 0 indicated the Donor baseline. **F** Communication strength relative to healthy donor. Bar plot showing total communication strength expressed as a percentage of the Donor baseline (100%, indicated by dashed line) for each functional category across the five CHD conditions. Y-axis represents relative strength (%)

Systematic analysis of cardiac ligand–receptor interactions revealed profound, phenotype-specific remodelling of intercellular communication networks across CHD (Fig. 3, Supplementary Table 4). Epicardial populations, involving EpiC and EpiL, were detectable but restricted to Donor and IF_HLHS groups at very low abundance and were analysed separately as a combined Epi population with results presented in Supplementary Fig. 3. Network architecture analysis distinguished that healthy donor hearts maintained balanced connectivity between EC and CM, and between CF and CM, whereas TOF and Neo_HLHS exhibited centralized EC to CM and CF to CM signalling, with EC and CF acting as dominant senders to passive CM. This notable pattern was absent in IF_HLHS, DCM, or HCM (Fig. 3A, B). IF_HLHS exhibited reduced density. In contrast, HCM showed the most fragmented network with weakened CF–CM and EC–CM coupling, while DCM preserved integrated connectivity with strengthened metabolic crosstalk (Fig. 3A, B). Together, these findings demonstrate that intercellular communication is profoundly reorganized across CHD, with each phenotype exhibiting a distinct signalling architecture.

Analysis of the top 25 cell–cell interactions (Fig. 3 C, Supplementary Table 5) highlighted that PTPRM–PTPRM homophilic signalling was predominant, with disease samples showing 1.5- to 2-fold increases versus healthy donors (Donor: ~2.5; TOF: ~4.5; DCM: ~4.3; Neo_HLHS: ~4.5; IF_HLHS: ~3.6). Similar increases occurred for NEGR1–NEGR1 and NCAM1–NCAM1, particularly in TOF and Neo_HLHS and DCM. Furthermore, homophilic adhesion molecules were the most consistently upregulated category across CHD types, particularly significantly elevated CD99–CD99 in Neo_HLHS (*P* < 0.05), or THBS1-CD36 in Neo_HLHS (*P* < 0.01). In addition, ECM–receptor interactions such as COL4A1-CD44 and COL4A2-CD44 were significantly increased in Neo_HLHS (*P* < 0.05) (Supplementary Table 5).

Ligand-receptor resolution and pathway-level quantification (Fig. 3D-F) revealed Neo_HLHS showed the strongest hyperactivation, with growth factor signalling (IGF, FGF) reaching 400–480% and ECM structural pathways ~ 380% of healthy donor levels, whereas TOF exhibited moderate activation (300–370% for IGF/FGF, 100–200% for others), both were supported by upregulated IGF2–IGF1R/IGF2R and ECM interactions (COL4A2/FN1–SDC4), indicating aggressive developmental compensation. IF_HLHS displayed global suppression near baseline, reflecting a hypoplastic communication profile with selective Notch and ECM pathway downregulation. DCM showed selective metabolic enhancement (~ 350%) and moderate growth factor upregulation, consistent with balanced metabolic and growth factor ligand-receptor enhancement. In contrast, HCM maintained near-baseline activity (80–150%) but showed severe morphogen suppression (~ 20% of Donor) and moderate Notch downregulation (~ 80%). Paradoxically, HCM upregulated CD99–CD99 junction signalling yet suppressed ECM adhesive (COL6A2/THBS3–SDC4) and Notch pathways (JAG2/DLL1–NOTCH2), suggesting profound loss of developmental homeostasis underlying its irreversible progression.

### Novel mechanistic insights and translational outlook

Building upon Hill et al.‘s (2022) transcriptomic framework, our communication-focused reanalysis reveals that homophilic adhesion genes (*PTPRM*, *NEGR1*, *NCAM1*, *CD99*) constitute the dominant remodelling signature across all CHD phenotypes, with 1.5- to 2-fold elevations over healthy donors, which could be considered as primary therapeutic targets for stabilizing tissue architecture in CHD through functional validation in disease-relevant models. Beyond this convergent signature, disease-specific communication patterns suggest distinct therapeutic entry points for each CHD subtypes. In structural CHD (TOF, Neo_HLHS) centralized EC to CM signalling with marked growth factor (IGF and FGF) hyperactivation (400–480% of Donor) may represent compensatory remodelling in malformed hearts. Whether targeted modulation of these growth factor pathways could prevent maladaptive remodelling and preserve cardiac function in these patients is an important question for future experimental investigation.

In DCM, selective metabolic enhancement (~ 350%) with preserved network connectivity points toward adaptive stress responses that may be amenable to metabolic intervention, including mitochondrial-targeted agents, ketone supplementation [72], or SGLT2 inhibitors [73]. These pharmaceuticals represent promising candidates worthy of evaluation in DCM specific models, with the goal of sustaining adaptive responses and delaying heart failure progression.

The most striking finding is the profound morphogen suppression observed in HCM (~ 20% of Donor), accompanied by widespread network fragmentation. This pattern is consistent with the limited regenerative capacity and progressive clinical course characteristic of HCM, as morphogen pathways govern regenerative capacity and developmental homeostasis [74]. Future therapeutic development for HCM may consider investigating modulation of morphogen pathways, such as through recombinant BMP or Wnt agonists, small molecule Hedgehog activators, or regenerative strategies designed to enhance morphogen signalling.

## Limitation and future perspectives

The current analysis infers signalling from transcript-level co-expression, which may not fully capture protein abundance, ligand availability, receptor activation, or post-transcriptional regulation. Therefore, functional validation using orthogonal approaches such as co-culture assays or spatial proteomics will be required to confirm active ligand-receptor engagement. Additionally, the reanalysed dataset contains a small number of patients with considerable age heterogeneity between groups, and notably Neo_HLHS and HCM are each represented by a single patient, limiting statistical power and potentially confounding condition-specific findings. Gender imbalance across groups should also be considered, as IF_HLHS and DCM contain only males while HCM only a female. Moreover, the study relies on a single cross-sectional dataset without longitudinal sampling, preventing assessment of temporal dynamics or causal progression of signalling alterations. Therefore, multi-cohort validation and integration with independent datasets will be essential to evaluate reproducibility and generalisability.

Future studies integrating latest omics approaches such as single-cell assay for transposase-accessible chromatin sequencing (scATAC-seq) and spatial transcriptomics [26, 29] may help contextualize communication networks within chromatin accessibility and tissue architecture landscapes. When combined with proteomic and functional validation, these multimodal approaches could provide a more comprehensive understanding of how cell-cell signalling relates to structural remodelling and disease progression.

## Conclusions

Single-cell technologies have provided unprecedented insights into CHD pathogenesis, revealing cellular heterogeneity and molecular mechanisms underlying diverse disease subtypes. Building upon these advances, our reanalysis of the Hill et al. dataset systematically mapped intercellular communication networks across multiple CHD conditions, demonstrating that each subtype exhibits a distinct signalling architecture. These findings highlight that CHD pathogenesis involves not only cell-autonomous transcriptional defects but also profound disruptions in intercellular communication. The identification of both shared and disease-specific signalling alterations may provide a foundation for the development of precision therapeutic strategies tailored to individual CHD subtypes, which will require functional validation using patient-derived models and replication across independent cohorts before clinical translation. Future studies integrating advanced approaches such as scATAC-seq, spatial transcriptomics, and proteomics will be essential to contextualize these communication networks within their native tissue architecture and chromatin landscapes. Coupled with functional validation using patient-derived models and longitudinal clinical studies, these multimodal approaches will likely advance our mechanistic understanding and accelerate translation toward precision medicine for CHD patients.

## Supplementary Information

Supplementary Material 1.

Supplementary Material 2.

Supplementary Material 3.

Supplementary Material 4.

Supplementary Material 5..

Supplementary Material 6

Supplementary Material 7.

Supplementary Material 8.

Supplementary Material 9.

Supplementary Material 10.

Supplementary Material 11.

## Data Availability

The Hill et al. dataset (15) is available on: https:/www.ncbi.nlm.nih.gov/geo/query/acc.cgi? acc=GSE203275. The data analysis pipeline flowchart used in this manuscript is shown in Supplementary Figure 1. The analysis code is available from the authors upon request.

## Abbreviations

Adipo: Adipocyte
ATAC-seq: Assay for transposase-accessible chromatin sequencing
AVSD: Atrioventricular septal defect
BMP: Bone morphogenetic protein
bulk RNA-seq: Bulk RNA sequencing
CF: Cardiac fibroblast
CHD: Congenital heart disease
CM: Cardiomyocyte
CTM: Conotruncal heart malformation
DCM: Dilated cardiomyopathy
DEG: Differentially expressed gene
EC: Endothelial cell
ECM: Extracellular matrix
EndMT: Endothelial-to-mesenchymal transition
ENDOC: Endocardial cell
EpiC: Epicardial cell
EpiL: Epithelial-like cell
FGF: Fibroblast growth factor
FHF: First heart field
HCM: Hypertrophic cardiomyopathy
HLHS: Hypoplastic left heart syndrome
HRHS: Hypoplastic right heart syndrome
IF_HLHS: Infant hypoplastic left heart syndrome
IGF: Insulin-like growth factor
iPSC: Induced pluripotent stem cell
LEC: Lymphatic endothelial cell
log₂FC: Log base-2 fold change
Mac: Macrophage
Mast: Mast cell
MERFISH ST: Multiplexed error-robust fluorescence in situ hybridization spatial transcriptomics
Multiome: Combined snRNA-seq + snATAC-seq (simultaneous profiling from the same nuclei)
NCC: Neural crest cell
Neo_HLHS: Neonatal hypoplastic left heart syndrome
NF-κB: Nuclear factor kappa-light-chain-enhancer of activated B cells
PCA: Principal component analysis
PeriC: Pericyte
PGC1α: Peroxisome proliferator-activated receptor gamma coactivator 1-alpha
scATAC-seq: Single-cell assay for transposase-accessible chromatin sequencing
scRNA-seq: Single-cell RNA sequencing
SGLT2: Sodium-glucose cotransporter 2
SHF: Second heart field
SMC: Smooth muscle cell
snATAC-seq: Single-nucleus assay for transposase-accessible chromatin sequencing
snRNA-seq: Single-nucleus RNA sequencing
ST: Spatial transcriptomics
TF: Transcription factor
TGF-β: Transforming growth factor beta
TOF: Tetralogy of fallot
UMAP: Uniform manifold approximation and projection
Visium ST: Visium spatial transcriptomics (10x genomics platform)
VSD: Ventricular septal defect
wks: Weeks (gestational age)
yrs: Years (age)
