# Supplementary figures and images for: Single-cell omics uncovers novel pathological mechanisms and therapeutic targets for congenital heart diseases: insights from integrated intercellular communication analysis
Source: Stem Cell Res Ther. 2026 Apr 16;17:198. doi: 10.1186/s13287-026-05015-3 (PMC13202810; doi:10.1186/s13287-026-05015-3)

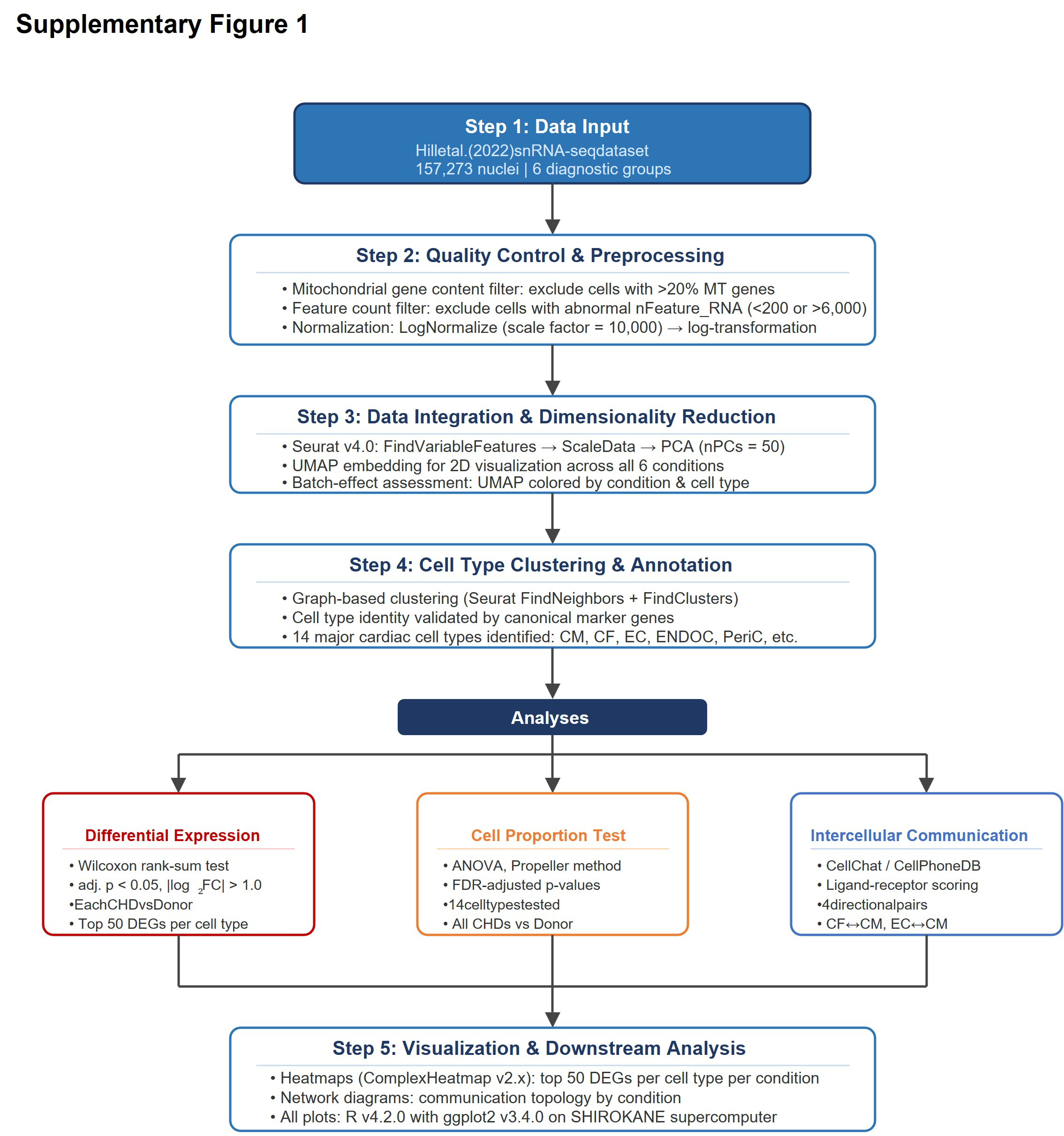

Supplement: Supplementary file 1 — Supplementary Material 1. [file 13287_2026_5015_MOESM1_ESM.tif]

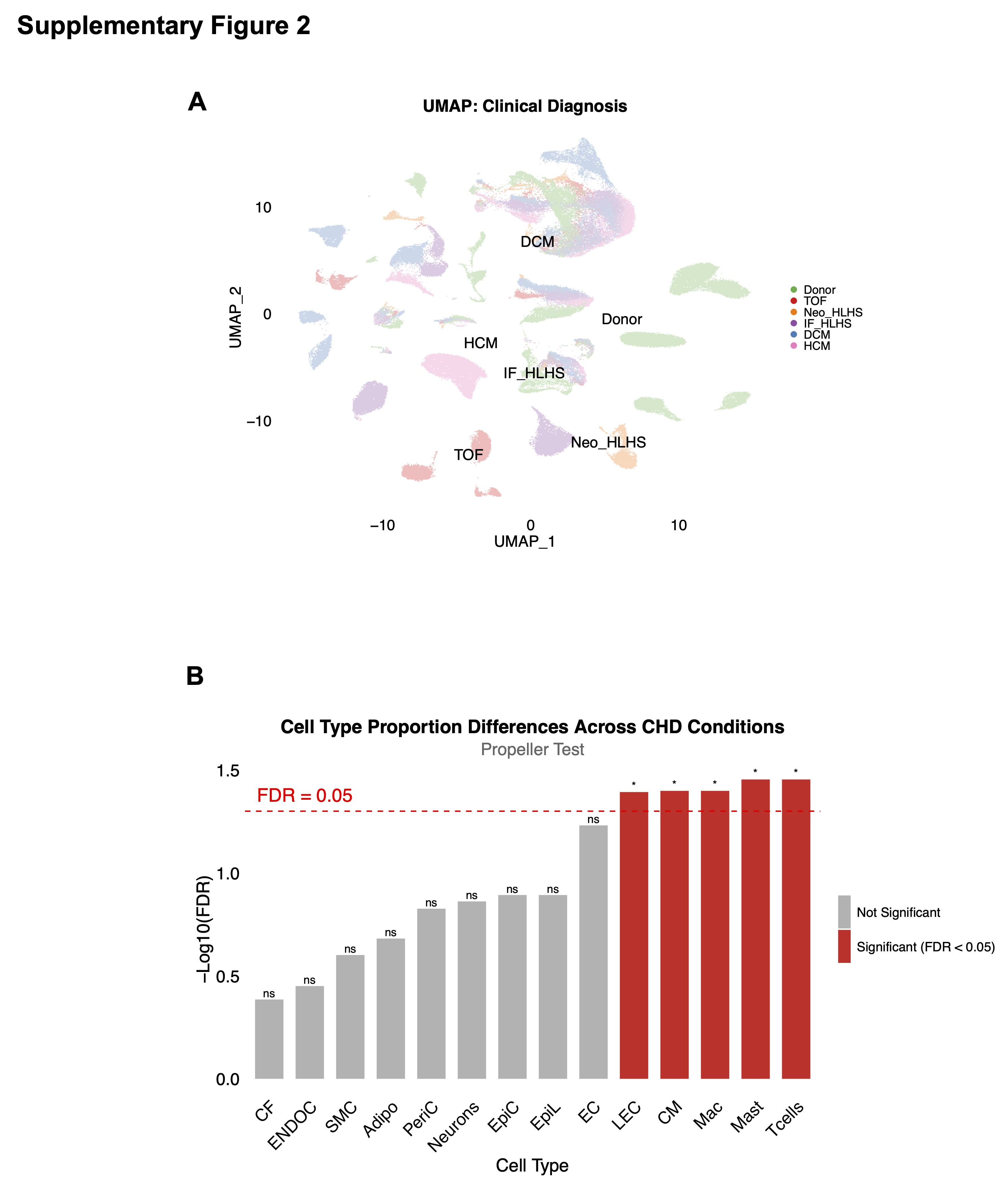

Supplement: Supplementary file 2 — Supplementary Material 2. [file 13287_2026_5015_MOESM2_ESM.tiff]

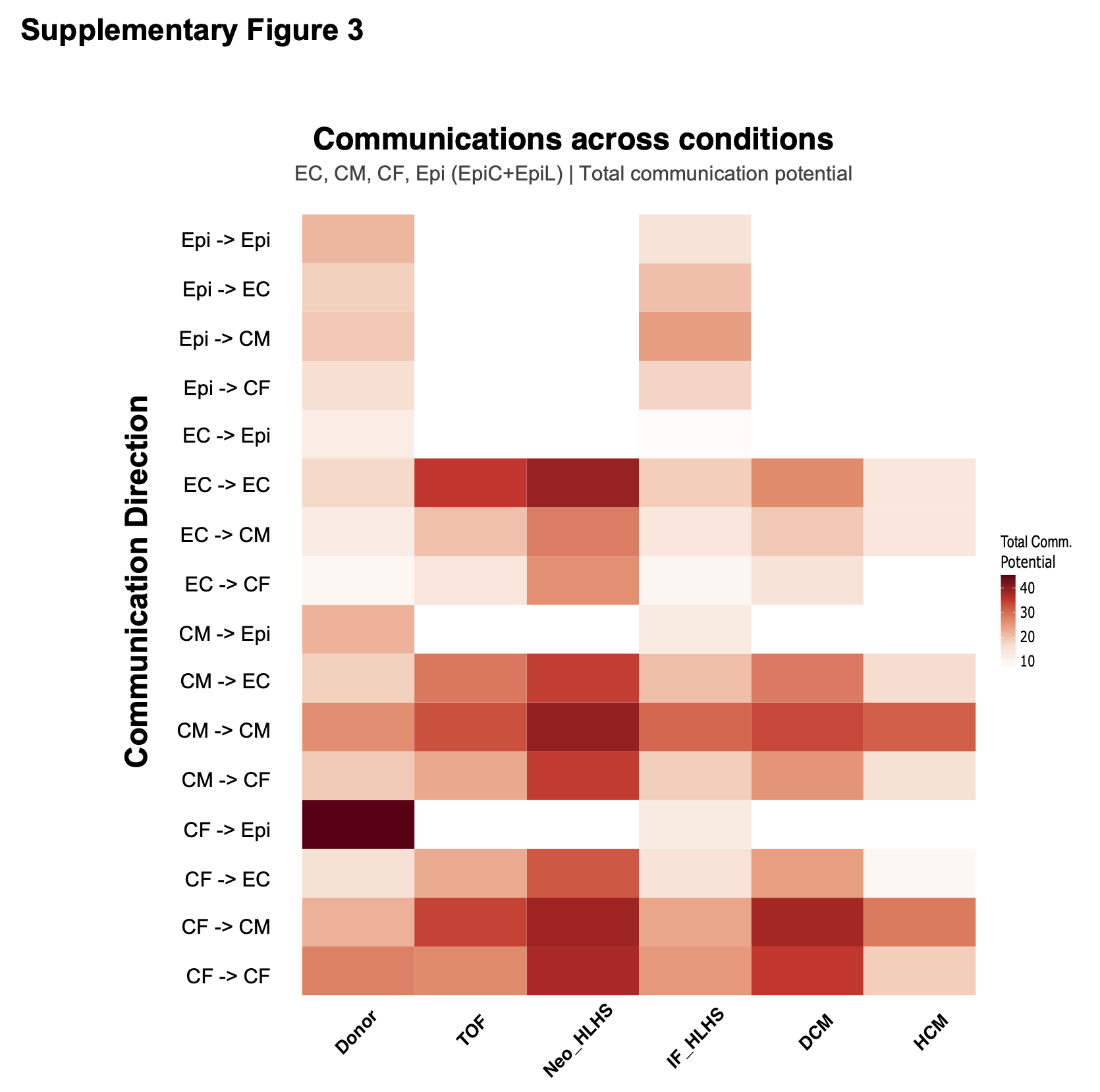

Supplement: Supplementary file 3 — Supplementary Material 3. [file 13287_2026_5015_MOESM3_ESM.tiff]
