# Supplementary material for: Single-cell omics uncovers novel pathological mechanisms and therapeutic targets for congenital heart diseases: insights from integrated intercellular communication analysis
Source: Stem Cell Res Ther. 2026 Apr 16;17:198. doi: 10.1186/s13287-026-05015-3 (PMC13202810; doi:10.1186/s13287-026-05015-3)
Supplement: Supplementary file 4 — Supplementary Material 4. [file 13287_2026_5015_MOESM4_ESM.docx]

**Supplementary Figure Legends**

**Supplementary Figure 1. Data analysis pipeline flowchart.** Schematic overview of the computational workflow applied to the Hill et al. (2022) snRNA-seq dataset. The pipeline encompasses five sequential steps: (1) data input (GEO accession GSE203275; 157,273 nuclei across six diagnostic groups); (2) quality control and preprocessing (mitochondrial content filter >20%; abnormal Feature count filter <200 or >6,000; LogNormalize method, scale factor = 10,000); (3) dimensionality reduction (PCA followed by UMAP embedding); (4) cell type clustering and annotation (14 major cardiac cell types identified by canonical marker genes); and (5) parallel downstream analyses comprising differential expression analysis (Wilcoxon rank-sum test), cell type proportion testing, and intercellular communication analysis. All analyses were performed in R v4.2.0 on the SHIROKANE supercomputer (University of Tokyo).

**Supplementary Figure 2. Cell type proportion test results.**

**(A) UMAP visualization by clinical diagnosis.**UMAP embedding of all cells coloured by clinical conditions.

**(B) Cell type proportion test results (Propeller).** Bar plot showing −log₁₀(FDR) for each of the 14 major cardiac cell types from the Propeller test (Phipson et al., 2022), which applies a linear model framework to test for statistically significant differences in cell type proportions across all CHD conditions versus healthy donor controls, accounting for sample variability. Cell types with FDR < 0.05 are highlighted in red, indicating statistically significant proportion changes. The dashed line indicates the FDR = 0.05 significance threshold (−log₁₀ = 1.30). CM, Tcells, Mast, Mac and LEC reached statistical significance (FDR < 0.05), while EC was approaching significance (FDR = 0.059).

**Supplementary Figure 3.** Intercellular communication networks including epicardial cells.

Network diagrams showing intercellular communication strength across all six conditions (Donor, TOF, Neo_HLHS, IF_HLHS, DCM, HCM) for a combined epicardial-cell (Epi) population (EpiC + EpiL) alongside CM, CF, and EC. Epicardial cells were detectable primarily in Donor and IF_HLHS groups. In Donor hearts, Epi cells engaged in VEGFA–NRP1 and EDN1–EDNRA signalling with EC and CM respectively, consistent with roles in vascular tone regulation and epicardial–myocardial crosstalk under homeostatic conditions. In IF_HLHS, Epi-mediated communication was markedly attenuated relative to Donor, consistent with the severely reduced epicardial cell abundance. Node size represents cell type abundance; edge width represents communication strength (mean crosstalk potential).
